# Management of iatrogenic airway bleeding with flexible bronchoscopy: Evidence or experience-based?

**DOI:** 10.5578/tt.20239608

**Published:** 2023-12-04

**Authors:** Oğuz KARCIOĞLU, Ziya Toros SELÇUK

**Affiliations:** 1 Department of Chest Diseases, Hacettepe University Faculty of Medicine, Ankara, Türkiye

## Abstract

**ABSTRACT**

**
Management of iatrogenic airway bleeding with flexible bronchoscopy: Evidence or experience-based?
**

Iatrogenic bleeding during bronchoscopy may lead to early termination, insuf- ficient sample collection, decreased diagnostic accuracy, and even death. Unlike rigid bronchoscopy, the management of bleeding during flexible fibe- roptic bronchoscopy does not allow the use of methods such as cautery, direct pressure, etc. and is usually limited to the application of liquids. The management of endobronchial bleeding usually depends on two main mec- hanisms: 1) vasoconstriction; 2) enhancing coagulation to form fibrin clots. The data on cold saline, the most widely recognized agent, is based on case reports and the experience of centers, not randomized controlled trials. Vasoconstrictor agents consist of adrenaline, vasopressin analogues, pheny- lephrine, and xylometazoline hydrochloride. However, there are only a limited number of randomized controlled trials on adrenaline, and information on the remaining substances is limited to retrospective studies, case reports, and expert opinions. The endobronchial administration of tranexamic acid, which inhibits fibrin degradation, has been the subject of very few studies. Despite its documented efficacy, information regarding its dosage, frequency of use, and safety is lacking. Although Ankaferd Blood Stopper, which binds eryt- hrocytes to the vascular endothelium, has been shown to be effective in controlling bleeding related to dental procedures, the gastrointestinal tract, and operations, only one retrospective study found it to be effective against endobronchial bleeding that could not be controlled with cold saline and adrenaline. Although there are a variety of agents that centers use in their routine procedures, there is not yet a consensus on the efficacy, dose, frequ- ency, and safety of any of them.

**Key words:** Hemorrhage; intervention; bronchoscopy; mucosa; bronchi

**ÖZ**

**
Fleksibl bronkoskopi ile iyatrojenik hava yolu kanamasının yönetimi: Kanıta mı, deneyime mi dayalı?
**

Bronkoskopi sırasında iyatrojenik kanama işlemin erken sonlandırılmasına,

yetersiz örneklemeye, tanısal doğruluğun azalmasına yol açabileceği gibi ölümle de sonuçlanabilir. Rijit bronkoskopinin aksine, fleksibl fiberoptik bron-

koskopi sırasında kanamanın yönetimi koter, doğrudan basınç vb. yöntemlerin kullanılmasına izin vermez ve genellikle sıvı uygulama- sıyla sınırlıdır. Endobronşiyal kanamanın yönetimi genellikle iki ana mekanizmaya bağlıdır: 1) vazokonstriksiyon, 2) fibrin formasyonu için pıhtılaşmanın sağlanması. En yaygın olarak bilinen ajan olan soğuk salin ile ilgili veriler, randomize kontrollü çalışmalardan çok vaka raporlarına ve merkezlerin deneyimlerine dayanmaktadır. Vazokonstriktör ajanlar adrenalin, vazopressin analogları, fenilefrin ve ksilometazolin hidroklorürden oluşur. Yalnızca adrenalinle ilgili sınırlı sayıda randomize kontrollü çalışma olup geri kalan maddelerle ilgili bilgiler retrospektif çalışmalar, vaka raporları ve uzman görüşleriyle sınırlıdır. Fibrin yıkımını inhibe eden traneksamik asidin endobronşiyal uygulaması çok az çalışmaya konu olmuştur. Etkinliği gösterilmiş olmasına rağmen, dozajı, kullanım sıklığı ve güvenliği ile ilgili bilgiler yetersizdir. Eritrositleri vasküler endotele bağlayan Ankaferd Blood Stopper’ın diş prosedürleri, gastrointestinal sistem ve ameliyatlarla ilgili kanamaların kontrolünde etkili olduğu gösterilmiş olsa da, sadece bir retrospektif çalışmada soğuk salin ve adre- nalin ile kontrol edilemeyen endobronşiyal kanamalara karşı etkili olduğu bulunmuştur. Merkezlerin rutin prosedürlerinde kullandık- ları çeşitli ajanlar olmasına rağmen, hiçbirinin etkinliği, dozu, sıklığı ve güvenliği konusunda henüz bir fikir birliği yoktur.

**Anahtar kelimeler:** *Kanama; girişimsel; bronkoskopi; mukoza; bronş
*

**INTRODUCTION**

Bronchoscopy, a traditional yet indispensable instrument for chest clinicians, remains one of the most important diagnostic tools for both pulmonary and mediastinal diseases. Both the image quality and maneuverability of the devices improved with advances in technology. Moreover, combination devices such as convex probe endobronchial ultrasonography (c-EBUS), navigation bronchoscopes, radial probe endobronchial ultrasonography (r-EBUS), cryoprobes, electrocautery, and lasers enabled sampling and management of even difficult- to-reach mediastinal structures and periphery of the lung.

However, there is a complication; namely bleeding, which may be controlled by suction alone or potentially lead to early termination of the procedure, inadequate sampling, decreased diagnostic accuracy, and even death (1,2). The severity of the bleeding differs depending on factors associated with patients, target lesions, and type of procedures. Pulmonary hypertension, coagulation disorders, use of anti- coagulant or anti-platelet drugs, low platelet counts (<50.000/mm^3^), uremia, immuncompromisation are related with increased risk of bleeding with bronchoscopy (3-6). It has been reported that vascular, adjacent to main vessels, centrally located, and solid peripheral lesions are more likely to be associated with hemorrhage (2,7). Because of the hypervascularisation of carcinoids and endobronchial metastasis, as well as the dual blood supply (both bronchial and pulmonary arteries) of peripheral lung lesions, these may also be associated with high risk of bleeding after bronchoscopy (8,9). Type of procedure is also critical to predict the bleeding risk. Whereas the risk of hemorrhage from observation alone or bronchial lavage is negligible even in high-

risk patients (4,10), it increases for brushing, normal mucosal biopsy, conventional transbronchial needle aspiration (c-TBNA), EBUS-TBNA, endobronchial biopsy of the tumoral lesions, transbronchial lung biopsy (TBLB), and transbronchial cryobiopsy (TBLC) of peripheral lesions (2,9-14).

Managing the bleeding during bronchoscopic procedures differs between flexible fiberoptic bronchoscopy (FFB) and rigid bronchoscopy. Rigid bronchoscopy has its own advantages. Firstly, it allows an infinite number of entries and exits, whether using rigid instruments or FFB within a rigid tube, as well as the ability to clean the tip of the instruments to improve visibility. Bronchoscopists can apply tampons to the bleeding site or use balloon tampons, endobronchial stents or valves, argon plasma coagulation, or liquids such as cold saline, adrenaline, and tranexamic acid directly or via FFB (15,16). However, in the vast majority of centers, rigid bronchoscopy is either not preferred or cannot be performed due to the lack of adequate personnel and equipment. Handling the bleeding with FFB is more difficult and sometimes frightening. It is impossible to move it in and out of the bronchoscope repeatedly for cleaning or using equipment such as tamponades, argon, or cautery. It does not offer sufficient suction for significant volumes or enough pressure to put on the bleeding site. Therefore, liquid agents remain to be key tools to manage the bleeding via FFB. This review aimed to discuss each of these agents in detail.

## Cold Saline

The concept of cold saline usage stems theoretically from the knowledge of the vasoconstrictor effect of cold and practically from case series reporting huge amounts (160-2500 mL) of cold saline administered

via rigid bronchoscopy for massive hemoptysis (17-19). The term “cold”, or “ice-cold” saline refers to 4 ºC. Although it is widely known and included in guideline suggestions, there is no evidence-based recommendation on the amount, number, and duration of cold saline applications (4). Our knowledge of the use of cold saline with FFB in small amounts is also based on case reports and expert opinion and not randomized controlled trials. Among the very few complications reported are transient sinus bradycardia and complete heart block (18,20).

## Adrenaline

Adrenaline (epinephrin) is the most widely studied agent for iatrogenic endobronchial bleeding. As a vasoconstrictor, it may aid to maintain hemostasis by reducing blood flow to the bleeding vessel. The information about the use of adrenaline for iatrogenic bleeding is from case series and center experiences rather than randomized controlled trials. The first comprehensive report from the Cleveland Clinic revealed that bleeding occurred in 58 cases after bronchoscopic interventions between 1981 and 1989, of which 39 (67.2%) resolved spontaneously

and 19 (32.8%) required additional interventions. They preferred intrabronchial instillation of 1:1000 adrenaline (1:10 diluted with normal saline) up to three times as needed. Although the bleeding stopped without any adverse effects, they cautioned that it should be used carefully due to potential side effects including hypertension, coronary vasospasm, and arrhythmia, particularly in high-risk individuals (6).

Studies regarding the use of adrenaline in cases of bronchoscopy-related bleeding primarily aimed to evaluate the efficacy of adrenaline in various doses (2,6,21). A new study comparing the effectiveness of tranexamic acid (TXA) and adrenaline has reported the use of 2 mL of 1:10000 adrenaline solution per application as a usual institution protocol and 1.8 ±

0.8 applications to manage bleeding. They have concluded that there was no difference between TXA and adrenaline in terms of bleeding control (22). In a comparable trial, Fekri et al. have used 1 mg of 1:20 diluted adrenaline solutions to control the bleeding. Mean applications to control the bleeding were 1.8 ± 0.74, and there was no difference between TXA and adrenaline (23). Therefore, there is a lack of information regarding the precise indications of endobronchial adrenaline, as well as the optimal

and maximal doses (24). The current British Thoracic Society (BTS) Guideline for Flexible Broncoshopy in Adults suggests a dose of 5-10 mL of 1:10000 adrenaline can be used alternatively with cold saline (4).

Unlike other agents, the route of administration must be considered when using adrenaline. Although potential adverse effects are known, they are more pronounced when adrenaline is administered peripherally. Steinfort et al. have reported that ventricular fibrillation developed in two patients after the administration of adrenaline with guide sheath due to uncontrolled bleeding despite suction following TBLB (25). The use of adrenaline for bleeding originating from the periphery of the lung may cause coronary vasospasm even in low doses (26). Although older reports advocated that there is no upper limit for endobronchial adrenaline use, it should be known that bleeding from the lung periphery is not the same as directly visible endobronchial bleeding. The adverse effects are related to systemic absorption (24). Administering adrenaline with a guide sheath or diluting it with a larger volume of saline and then applying it with a wedge maneuver causes adrenaline to reach a greater portion of the alveolocapillary membrane, thereby enhancing systemic passage. Despite the fact that the majority of centers continue to use it, there are some advocates for abandoning endobronchial administration due to the possibility of severe adverse effects (27).

## Tranexamic Acid

As a synthetic lysine derivative, tranexamic acid (TXA) inhibits the plasminogen to plasmin conversion by blocking the lysine binding sites of plasminogen, which in turn causes the inhibition of fibrin clot degradation (28). In contrast to other options, TXA has a broader range of indications for bleeding originating from the lungs and multiple administration routes. It has been shown to be effective in hemoptysis associated with various lung diseases, including cystic fibrosis, malignancies, etc., when used intravenously (IV), orally, or by nebulization (29-31).

The knowledge about the utility of TXA against bleeding originates from studies of trauma- or surgery-related bleedings (32,33), but there are no randomized studies concerning endobronchial bleeding. Solomonov et al. have published the first two cases of endobronchial bleeding (one

spontaneous and one following TBLB) controlled by endobronchial administration of TXA via bronchoscopy (34). A larger study has reported endobronchial TXA application was successful in the management of iatrogenic bleeding (15 airways, five parenchyma) that could not be stopped despite the administration of cold saline and adrenaline, with no adverse effects confirmed (35). Two aforementioned trials comparing TXA and adrenaline have also concluded that endobronchial use of TXA is effective in iatrogenic bleeding (22,23).

As an antifibrinolytic drug, theoretically, TXA is thought to be associated with an increased risk of thrombosis. While the systemic administration of tranexamic acid has been linked to an elevated risk of venous embolism, there is currently no evidence suggesting that endobronchial administration is associated with an increased risk of thrombosis, including venous embolism (36-38).

## Xylometazoline Hydrochloride

Xylometazoline hydrochloride (XH) is a widely used nasal topical vasoconstrictor to relieve symptoms of rhinitis and sinusitis. Its effects on hemorrhage were particularly studied in nasotracheal intubation. El-Seify et al. have argued that administering XH prior to nasotracheal intubation reduces both the incidence and severity of bleeding (39). The only study on the use of XH in bronchoscopy investigated the nasal use of XH before bronchoscopy but not bleeding during bronchoscopy (40). Although there is no further research on its effects, many centers use XH in the case of bleeding during bronchoscopy.

## Ankaferd Blood Stopper

Ankaferd Blood Stopper (ABS) is a relatively novel agent that has been used historically but has also been proven molecularly. Its primary mechanism of action is to form a protein network between erythrocytes and the vascular endothelium (41). Although the efficacy of ABS has been demonstrated in many areas, including external injuries due to trauma (42), dental procedures (43), and post- operative bleeding (44), the notion that it can be used to manage bleeding from the airways is primarily based on data from gastrointestinal system (GIS) bleeding. Numerous clinical studies have demonstrated its efficacy in both non-traumatic

(variceal, tumor) (45) and traumatic (interventional procedures: biopsy, polipectomy) (46) GIS bleeding.

There is a paucity of information regarding the effectiveness of ABS on lung and airway bleeding. Uzun et al. have concluded in the sole investigation involving a significant number of patients that ABS was efficacious for both spontaneous and biopsy- induced hemoptysis that could not be stopped with cold saline and adrenaline. They have argued that ABS allowed the prompt management of malignant and benign airway or pulmonary bleeding without delay (47). It has also been reported to be effective in a patient with Mounhier Kuhn syndrome who presented with massive hemoptysis and could not be controlled with cold saline, adrenaline, and tranexamic acid (48).

From our unpublished experience with bleedings after bronchoscopic biopsy that could not be stopped with cold saline and adrenaline, we observed that ABS is highly effective and instantly stops bleeding upon contact with the bleeding site. Similar to the aforementioned publications, we apply it in 2 mL doses that may be repeated as necessary. ABS has the disadvantage of causing clotting in all areas of contact with blood, including the working channel and tip of the bronchoscope. This can lead to image distortion and blockage of the working channel, forcing the bronchoscope to be removed. We believe administration with a catheter in distance to the distal end of the bronchoscope will be effective in preventing this complication.

## Vasopressin Analogues

Vasopressin (antidiuretic hormone) analogues, including terlipressin and ornipressin, have been frequently used for GI bleeding. There are two studies about the endobronchial use of vasopressin analogues (49,50). The relatively older study, published in 1989, primarily aimed to compare the intravenous versus endobronchial use of terlipressin (glypressin) in terms of levels and clinical effects during endobronchial bleeding. They stated that bleeding stopped and mucosa turned pallor immediately after the application via either route (49). Tuller C. and colleagues studied the adverse effects of two vasopressin analogs, terlipressin and ornipressin, during endobronchial bleeding in a study with a similar design. Both of the drugs were

reported to be clinically efficacious on endobronchial bleeding after mucosal biopsy, transbronchial biopsy, and needle aspiration, and there were no clinically significant differences in regard to adverse effects (50). Although they are not commonly used clinically to our knowledge, it has been reported that they can be used in cases of uncontrolled bleeding following suction and cold saline (9).

## Phenylephrine

Normally, phenylephrine is administered prior to bronchoscopy to widen the nasal passage and make it easier to move the scope (9,51). Although endobronchial phenylephrine is also widely used in many centers, knowledge about its efficacy is based not on randomized controlled trials but on center experiences. The sole research investigating the safety but not efficacy of phenylephrine in endobronchial bleeding was a retrospective study that encompassed not only iatrogenic bleeding but also the administration of phenylephrine in instances of spontaneous bleeding and the prophylactic use of phenylephrine in cases of high-risk bleeding. In all procedures, phenylephrine was administered through the working channel with a total volume of

6.5 ± 10.6 mL (100 mcg/mL). Although a statistically significant increase in mean blood pressure during the procedure compared to baseline has been reported, there were no life-threatening side effects (52).

The CHEST consensus statement aimed at defining bleeding after a posttransbronchial biopsy mentioned phenylephrine; however, there was no further information or reference to endobronchial administration (53).

## CONCLUSION

While most bleedings during fiberoptic bronchoscopy are often resolved without intervention, bronchoscopists still have significant challenges in managing cases of severe and uncontrollable bleeding. Although many centers have local practices such as the use of cold saline, vasoconstrictors, tranexamic acid, or other novel products, there is still no consensus (Table 1). The fact that it is a life- threatening complication that the bronchoscopist aims to control as soon as possible makes it difficult to perform prospective randomized controlled studies.

## CONFLICT of INTEREST

The authors declare that they have no conflict of interest.

## AUTHORSHIP CONTRIBUTIONS

Concept/Design: OK Analysis/Interpretation: ZTS Data acqusition: OK Writing: OK, ZTS

Clinical Revision: ZTS Final Approval: OK, ZTS

**Table:** 

**Table 1.** List of liquids used through the flexible bronchoscope to control bleeding				
**Product**	**Mechanism of action**	**Used doses**	**Side effect**	**Reference**
Cold saline	Vasoconstriction	5-2500 mL	Arrhytmia	4, 18, 19, 20
Adrenaline	Vasoconstriction	1-10 mL of 1:10000	Arrhytmia, coronary	2, 6, 22-27
			vasospasm	
Tranexamic acid	Anti-fibrinolysis	100-1500 mg	Not reported	22, 23, 28, 34-36
Xylometazoline hydro-	Vasoconstriction	Not reported	Not reported	37
chloride				
Ankaferd Blood Stopper	Enhancing protein net-	2-4 mL	Blockage in the work-	39, 45, 46
	work between erythro-		ing channel and	
	cytes and vascular		decreased visual quali-	
	endothelium		ty	
Vasopressin analogues	Vasoconstriction	Terlipressin 1 mg	Not reported	9, 47, 48
		Ornipressin 5 IU		
Phenylephrine	Vasoconstriction	650-1600 mcg	MAP elevation	9, 49-51
MAP: Mean arterial pressure, mL: Mililiters, mg: Miligrams, mcg: Micrograms, IU: International unit.				

## REFERENCES

1. *
  Herth FJF. Bronchoscopy and bleeding risk. Eur Respir Rev 2017; 26(145): 170052. https://doi. org/10.1183/16000617.0052-2017
  *
2. *
  Muthu V, Ram B, Sehgal IS, Dhooria S, Prasad KT, Bal A, et al. Predictors of severe bleeding during endobronchial biopsy. J Bronchology Interv Pulmonol 2019; 26(4): 273- 9. https://doi.org/10.1097/LBR.0000000000000593
  *
3. *
  Takashima Y, Shinagawa N, Morinaga D, Nakamura J, Furuta M, Shoji T, et al. Risk of bleeding associated with transbronchial biopsy using flexible bronchoscopy in patients with echocardiographic or chest CT evidence of pulmonary hypertension. BMC Pulmonary Medicine 2022; 22(1): 449. https://doi.org/10.1186/s12890-022- 02245-y
  *
4. *
  Du Rand I, Blaikley J, Booton R, Chaudhuri N, Gupta V, Khalid S, et al. British Thoracic Society Guideline for Diagnostic Flexible Bronchoscopy in Adults: Accredited by NICE. Thorax 2013; 68(Suppl 1): i1-i44. https://doi. org/10.1136/thoraxjnl-2013-203618
  *
5. *
  Ernst A, Eberhardt R, Wahidi M, Becker HD, Herth FJ. Effect of routine clopidogrel use on bleeding complica- tions after transbronchial biopsy in humans. Chest 2006; 129(3): 734-7. https://doi.org/10.1378/chest.129.3.734
  *
6. *
  Cordasco EM, Mehta AC, Ahmad M. Bronchoscopically induced bleeding: A summary of nine years’ Cleveland Clinic experience and review of the literature. Chest 1991; 100(4): 1141-7. https://doi.org/10.1378/ chest.100.4.1141
  *
7. *
  Asano F, Aoe M, Ohsaki Y, Okada Y, Sasada S, Sato S, et al. Deaths and complications associated with respiratory endoscopy: A survey by the Japan Society for Respiratory Endoscopy in 2010. Respirology 2012; 17(3): 478-85. https://doi.org/10.1111/j.1440-1843.2011.02123.x
  *
8. *
  Wang S, Ye Q. Bleeding after endobronchial biopsy: Sometimes frightening, often safe, always careful. BMC Pulm Med 2023; 23(1): 35. https://doi.org/10.1186/ s12890-023-02331-9
  *
9. *
  Bernasconi M, Koegelenberg CF, Koutsokera A, Ogna A, Casutt A, Nicod L, et al. Iatrogenic bleeding during flexible bronchoscopy: Risk factors, prophylactic measures and management. ERJ Open Res 2017; 3(2): 00084-2016. https://doi.org/10.1183/23120541.00084-2016
  *
10. *
  Nandagopal L, Veeraputhiran M, Jain T, Soubani AO, Schiffer CA. Bronchoscopy can be done safely in patients with thrombocytopenia. Transfusion 2016; 56(2): 344-8. https://doi.org/10.1111/trf.13348
  *
11. *
  Holty JC, Kuschner WG, Gould MK. Accuracy of trans- bronchial needle aspiration for mediastinal staging of non- small cell lung cancer: A meta-analysis. Thorax 2005; 60(11): 949-55. https://doi.org/10.1136/ thx.2005.041525
  *
12. *
  Gu P, Zhao YZ, Jiang LY, Zhang W, Xin Y, Han BH. Endobronchial ultrasound-guided transbronchial needle aspiration for staging of lung cancer: A systematic review and meta-analysis. Eur J Cancer 2009; 45(8): 1389-96. https://doi.org/10.1016/j.ejca.2008.11.043
  *
13. *
  Gershman E, Fruchter O, Benjamin F, Nader AR, Rosengarten D, Rusanov V, et al. Safety of cryo-transbron- chial biopsy in diffuse lung diseases: Analysis of three hundred cases. Respiration 2015; 90(1): 40-6. https://doi. org/10.1159/000381921
  *
14. *
  Du Rand I, Barber P, Goldring J, Lewis R, Mandal S, Munavvar M, et al. British Thoracic Society Guideline for Advanced Diagnostic and Therapeutic Flexible Bronchoscopy in Adults. Thorax 2011; 66(Suppl 3): iii1- iii21. https://doi.org/10.1136/thoraxjnl-2011-200713
  *
15. *
  Batra H, Yarmus L. Indications and complications of rigid bronchoscopy. Expert Rev Respir Med 2018; 12(6): 509- 20. https://doi.org/10.1080/17476348.2018.1473037
  *
16. *
  Davidson K, Shojaee S. Managing massive hemoptysis. Chest 2020; 157(1): 77-88. https://doi.org/10.1016/j. chest.2019.07.012
  *
17. *
  Alba BK, Castellani JW, Charkoudian N. Cold‐induced cutaneous vasoconstriction in humans: Function, dysfunc- tion and the distinctly counterproductive. Exp Physiol 2019; 104(8): 1202-14. https://doi.org/10.1113/ EP087718*
18. *
  Conlan AA, Hurwitz SS. Management of massive haemop- tysis with the rigid bronchoscope and cold saline lavage. Thorax 1980; 35(12): 901-4. https://doi.org/10.1136/ thx.35.12.901
  *
19. *
  Marsico GA, Guimarães CA, Montessi J, Costa AMMd, Madeira L. Management of massive hemoptysis with the rigid bronchoscope and cold saline lavage. Jornal de Pneumologia 2003; 29: 280-6. https://doi.org/10.1590/ S0102-35862003000500006
  *
20. *
  Sagar AES, Cherian SV, Estrada-Y-Martin RM. Complete heart block following cold saline lavage during bronchos- copy. J Bronchology Interv Pulmonol 2017; 24(3): 41-2. https://doi.org/10.1097/LBR.0000000000000392
  *
21. *
  Wang S, Ye Q. Association between blood pressure and the risk of biopsy-induced endobronchial hemorrhage during bronchoscopy. BMC Pulm Med 2022; 22(1): 25. https://doi.org/10.1186/s12890-022-01822-5
  *
22. *
  Badovinac S, Glodić G, Sabol I, Džubur F, Makek MJ, Baričević D, et al. Tranexamic acid vs adrenaline for con- trolling iatrogenic bleeding during flexible bronchoscopy: A double-blind randomized controlled trial. Chest 2023; 163(4): 985-93. https://doi.org/10.1016/j. chest.2022.10.013
  *
23. *
  Fekri MS, Hashemi-Bajgani SM, Shafahi A, Zarshenas R. Comparing adrenaline with tranexamic acid to control acute endobronchial bleeding: A randomized controlled trial. Iran J Med Sci 2017; 42(2): 129-35.
  *
24. *
  Khoo KL, Lee P, Mehta AC. Endobronchial epinephrine: Confusion is in the air. Am J Respir Crit Care Med 2013; 187(10): 1137-8. https://doi.org/10.1164/rccm.201209- 1682LE
  *
25. *
  Steinfort DP, Herth FJ, Eberhardt R, Irving LB. Potentially fatal arrhythmia complicating endobronchial epinephrine for control of iatrogenic bleeding. Am J Respir Crit Care Med 2012; 185(9): 1028-30. https://doi.org/10.1164/ ajrccm.185.9.1028
  *
26. *
  Janjua M, Badshah A, Allen S. Epinephrine-induced ST elevation: A case of endobronchial topical epinephrine-in- duced coronary vasospasm. Heart 2009; 95(8): 656. https://doi.org/10.1136/hrt.2008.161646
  *
27. *
  Edell ES. Time for a change. J Bronchology Interv Pulmonol 2013; 20(3): 198-9. https://doi.org/10.1097/ LBR.0b013e3182a113a2
  *
28. *
  Cai J, Ribkoff J, Olson S, Raghunathan V, Al‐Samkari H, DeLoughery TG, et al. The many roles of tranexamic acid: An overview of the clinical indications for TXA in medical and surgical patients. Eur J Haematol 2020; 104(2): 79-87. https://doi.org/10.1111/ejh.13348
  *
29. *
  Bellam BL, Dhibar DP, Suri V, Sharma N, Varma SC, Malhotra S, et al. Efficacy of tranexamic acid in haemop- tysis: A randomized, controlled pilot study. Pulm Pharmacol Ther 2016; 40: 80-3. https://doi.org/10.1016/j. pupt.2016.07.006
  *
30. *
  Wand O, Guber E, Guber A, Shochet GE, Israeli-Shani L, Shitrit D. Inhaled tranexamic acid for hemoptysis treat- ment: A randomized controlled trial. Chest 2018; 154(6): 1379-84. https://doi.org/10.1016/j.chest.2018.09.026
  *
31. *
  Prutsky G, Domecq JP, Salazar CA, Accinelli R. Antifibrinolytic therapy to reduce haemoptysis from any cause. Cochrane Database Syst Rev 2016; 11(11): CD008711. https://doi.org/10.1002/14651858. CD008711.pub3
  *
32. *
  Roberts I, Shakur H, Coats T, Hunt B, Balogun E, Barnetson L, et al. The CRASH-2 trial: A randomised controlled trial and economic evaluation of the effects of tranexamic acid on death, vascular occlusive events and transfusion requirement in bleeding trauma patients. Health Technol Assess 2013; 17(10): 1-79. https://doi.org/10.3310/ hta17100
  *
33. *
  Myles PS, Smith JA, Forbes A, Silbert B, Jayarajah M, Painter T, et al. Tranexamic acid in patients undergoing coronary-artery surgery. N Engl J Med 2017; 376(2): 136- 48. https://doi.org/10.1056/NEJMoa1606424
  *
34. *
  Solomonov A, Fruchter O, Zuckerman T, Brenner B, Yigla
  *

M. Pulmonary hemorrhage: A novel mode of therapy. Respir Med 2009; 103(8): 1196-200. https://doi. org/10.1016/j.rmed.2009.02.004

35. *
  Márquez-Martín E, Vergara DG, Martín-Juan J, Flacón AR, Lopez-Campos JL, Rodríguez-Panadero F. Endobronchial administration of tranexamic acid for controlling pulmo- nary bleeding: A pilot study. J Bronchology Interv Pulmonol 2010; 17(2): 122-5. https://doi.org/10.1097/ LBR.0b013e3181dc8c17
  *
36. *
  Myers SP, Kutcher ME, Rosengart MR, Sperry JL, Peitzman AB, Brown JB, et al. Tranexamic acid administration is asso- ciated with an increased risk of posttraumatic venous thromboembolism. J Trauma Acute Care Surg 2019; 86(1): 20-7. https://doi.org/10.1097/TA.0000000000002061
  *
37. *
  Krivokuca I, Lammers JW. Recurrent pulmonary embolism associated with a hemostatic drug: Tranexamic acid. Clin Appl Thromb Hemost 2011; 17(1): 106-7. https://doi. org/10.1177/1076029609340902
  *
38. *
  Chornenki NLJ, Um KJ, Mendoza PA, Samienezhad A, Swarup V, Chai-Adisaksopha C, et al. Risk of venous and arterial thrombosis in non-surgical patients receiving sys- temic tranexamic acid: A systematic review and meta-anal- ysis. Thromb Res 2019; 179: 81-6. https://doi. org/10.1016/j.thromres.2019.05.003
  *
39. *
  El-Seify Z, Khattab A, Shaaban A, Metwalli O, Hassan H, Ajjoub L. Xylometazoline pretreatment reduces nasotra- cheal intubation-related epistaxis in paediatric dental sur- gery. Br J Anaesth 2010; 105(4): 501-5. https://doi. org/10.1093/bja/aeq205
  *
40. *
  Sryma P, Mittal S, Tiwari P, Mohan A, Hadda V, Guleria R, et al. Topical nasal xylometazoline for flexible bronchosco- py (VAIN): A randomized, double-blind, placebo-con- trolled trial. Respir Investig 2021; 59(3): 350-5. https:// doi.org/10.1016/j.resinv.2020.12.004
  *
41. *
  Haznedaroglu BZ, Beyazit Y, Walker SL, Haznedaroglu IC. Pleiotropic cellular, hemostatic, and biological actions of Ankaferd hemostat. Crit Rev Oncol Hematol 2012; 83(1): 21-34. https://doi.org/10.1016/j.critrevonc.2011.10.006
  *
42. *
  Koyuncu N. The effectiveness of Ankaferd Blood Stopper in the management of traumatic bleeding. Adv Ther 2019; 36(5): 1143-9. https://doi.org/10.1007/s12325-019- 00935-4
  *
43. *
  Çakarer S, Eyüpoğlu E, Günes ÇÖ, Küseoğlu BG, Berberoğlu HK, Keskin C. Evaluation of the hemostatic effects of Ankaferd Blood Stopper during dental extractions in patients on antithrombotic therapy. Clin Appl Thromb Hemost 2013; 19(1): 96-9. https://doi. org/10.1177/1076029611435836
  *
44. *
  Guler M, Maralcan G, Kul S, Baskonus I, Yilmaz M. The efficacy of Ankaferd Blood Stopper for the management of bleeding following total thyroidectomy. J Invest Surg 2011; 24(5): 205-10. https://doi.org/10.3109/08941939.2011. 586092
  *
45. *
  Garber A, Jang S. Novel therapeutic strategies in the man- agement of non-variceal upper gastrointestinal bleeding. Clin Endosc 2016; 49(5): 421-4. https://doi.org/10.5946/ ce.2016.110
  *
46. *
  Mourad FH, Leong RW. Role of hemostatic powders in the management of lower gastrointestinal bleeding: A review. J Gastroenterol Hepatol 2018; 33(8): 1445-53. https:// doi.org/10.1111/jgh.14114
  *
47. *
  Uzun O, Erkan L, Haznedaroglu IC. Effective management of hemoptysis via endobronchial application of Ankaferd hemostat. Arch Bronconeumol 2014; 50(9): 407-9. https://doi.org/10.1016/j.arbres.2013.12.007
  *
48. *
  Yazici O, Gulen ST, Ceylan E. The effect of Ankaferd Blood Stopper used for massive haemoptysis in a patient with mounier-kuhn syndrome: A case report. J Pak Med Assoc 2020; 70(4): 731-3. https://doi.org/10.5455/ JPMA.295533
  *
49. *
  Breuer H, Charchut S, Worth H, Trampisch H, Glanzer K. Endobronchial versus intravenous application of the vaso- pressin derivative glypressin during diagnostic bronchos- copy. Eur Respir J 1989; 2(3): 225-8. https://doi. org/10.1183/09031936.93.02030225
  *
50. *
  Tüller C, Tüller D, Tamm M, Brutsche MH. Hemodynamic effects of endobronchial application of ornipressin versus terlipressin. Respiration 2004; 71(4): 397-401. https:// doi.org/10.1159/000079646
  *
51. *
  Latorre F, Otter W, Kleemann P, Dick W, Jage J. Cocaine or phenylephrine/lignocaine for nasal fibreoptic intubation? Eur J Anaesthesiol 1996; 13(6): 577-81. https://doi. org/10.1046/j.1365-2346.1996.00015.x
  *
52. *
  Pu CY, Ivanick N. QA project: Hemodynamic safety of endobronchial administration of phenylephrine for con- trol of airway bleeding by bronchoscopy. Pulm Pharmacol Ther 2020; 64: 101961. https://doi.org/10.1016/j. pupt.2020.101961
  *
53. *
  Folch EE, Mahajan AK, Oberg CL, Maldonado F, Toloza E, Krimsky WS, et al. Standardized definitions of bleeding after transbronchial lung biopsy: A Delphi consensus statement from the Nashville Working Group. Chest 2020; 158(1): 393-400. https://doi.org/10.1016/j. chest.2020.01.036
  *

